# COPD anxiety and depression across GOLD stages

**DOI:** 10.6026/973206300222292

**Published:** 2026-04-30

**Authors:** Sourabh Jain, Satyendra Mishra, Mahendra Singh Chouhan, Sanjay Gupta, Talha Saad

**Affiliations:** 1 Department of Emergency Medicine, Bundelkhand Medical College, Sagar, Madhya Pradesh, India; 2 Department of Respiratory Medicine, Bundelkhand Medical College, Sagar, Madhya Pradesh, India; 3 Department of Pediatrics, Bundelkhand Medical College, Sagar, Madhya Pradesh, India; 4 Department of TB and Respiratory Diseases, Chief Medical Officer (Senior Administrative Grade), NITRD, Delhi, India

**Keywords:** Chronic Obstructive Pulmonary Disease (COPD), FEV_1_, Global Initiative for Chronic Obstructive Lung Disease (GOLD) stage, anxiety, depression

## Abstract

Chronic Obstructive Pulmonary Disease (COPD) often leads to anxiety and depression, adversely impacting patients' quality of life. Therefore, it is of interest to evaluate the prevalence and correlation of anxiety and depression among COPD patients categorized by the GOLD guidelines. Hence, a total of 160 clinically stable COPD patients were assessed for anxiety and depression using the Hindi translations of PHQ-9 and GAD-7 questionnaires. Spirometry was used for classifying COPD severity as per GOLD guidelines. Significant correlations were found between GOLD categories and levels of anxiety (Spearman's rho = 0.351, P<0.01) and depression (Spearman's rho = 0.415, P<0.01). Data shows significant correlation between COPD severity and psychological distress, underscoring the importance of integrated mental health care in COPD management.

## Background:

Chronic Obstructive Pulmonary Disease (COPD) is a progressive lung disease characterized by persistent respiratory symptoms and airflow limitation due to airway and/or alveolar abnormalities. It is primarily caused by exposure to noxious particles or gases, particularly from cigarette smoke. COPD is a major cause of morbidity and mortality worldwide, contributing significantly to healthcare costs and economic burden [1]. COPD is often accompanied by comorbid psychological conditions, particularly anxiety and depression, which further deteriorate patients' quality of life and clinical outcomes [2]. These psychological disorders are linked to increased hospitalizations, reduced treatment adherence and impaired participation in pulmonary rehabilitation programs. Mental health disorders, especially anxiety and depression, are among the most common yet under diagnosed comorbidities in patients with COPD [3]. Despite their substantial impact, anxiety and depression are frequently under diagnosed and undertreated in COPD patients [4]. The Global Initiative for Chronic Obstructive Lung Disease (GOLD) classification provides a standardized method for staging disease severity based on spirometric findings [5]. Evaluating the relationship between GOLD stages and the prevalence of anxiety and depression can offer valuable insights into the psychosocial impact of disease progression. Understanding this correlation is essential for adopting a holistic approach to COPD management, emphasizing not only pulmonary function but also mental well-being [6, 7]. Therefore, it is of interest to evaluate the prevalence of anxiety and depression in COPD patients categorized by the Global Initiative for Chronic Obstructive Lung Disease (GOLD) guidelines and to explore the correlation between disease severity and psychological distress.

## Methods:

This hospital-based cross-sectional observational study was conducted in the Department of Pulmonary Medicine at Bundelkhand Medical College, Sagar, Madhya Pradesh, India, over a 12-month period from January 2024 to December 2024. The study included clinically stable COPD patients aged 18 years and above who were diagnosed according to GOLD guidelines and were willing to participate after providing written informed consent. Patients were excluded if they had significant co-morbid conditions or uncontrolled chronic diseases, a prior diagnosis of psychiatric illness or were currently taking psychotropic medications, cognitive impairment or inability to complete questionnaires, or if they were unwilling to provide consent. A total of 160 COPD patients attending the OPD who met the eligibility criteria were enrolled and evaluated for anxiety and depression. Detailed demographic information including age, sex, smoking history and duration of illness, along with clinical parameters, was recorded using a structured proforma. Spirometry was performed using a calibrated spirometer in accordance with standard guidelines and American Thoracic Society standards and patients were categorized into GOLD stages I-IV based on post-bronchodilator FEV_1_ (%) values, where GOLD I (mild) was defined as FEV_1_ ≥ 80% predicted, GOLD II (moderate) as FEV_1_ 50-79% predicted, GOLD III (severe) as FEV_1_ 30-49% predicted and GOLD IV (very severe) as FEV_1_ < 30% predicted. Depression was assessed using the Patient Health Questionnaire-9 (PHQ-9), with scores corresponding to mild (5), moderate (10), moderately severe (15) and severe (20) depression, while anxiety was assessed using the Generalized Anxiety Disorder-7 (GAD-7), with scores indicating mild (5), moderate (10) and severe (15) anxiety; a cutoff score of 10 was used to identify generalized anxiety disorder. Ethical approval was obtained from the Institutional Ethics Committee, written informed consent was secured from all participants before enrollment and confidentiality of patient data was strictly maintained. Data were entered into Microsoft Excel and analyzed using SPSS version 24, with the association between GOLD stages and anxiety/depression assessed using the Chi-square test and correlation analysis performed using Pearson or Spearman correlation coefficients as appropriate, considering a p-value of <0.05 as statistically significant.

## Results:

Most patients belonged to GOLD category B (41.3%), followed by category D (28.1%). GOLD category C constituted the smallest proportion (7.5%) of the study population. Anxiety was present in 36.25% of COPD patients. The highest prevalence of anxiety was observed in GOLD category D (57.78%), indicating increased psychological burden with advancing disease severity. Depression was detected in 44.38% of the study population. GOLD category D showed the highest prevalence of depression (68.89%), suggesting a strong association between disease severity and depressive symptoms. Spearman's correlation analysis revealed a statistically significant weak positive correlation between GOLD category and anxiety scores (ρ = 0.351, P < 0.01) and a moderate positive correlation with depression scores (ρ = 0.415, P < 0.01). Table 1 (see PDF) shows the baseline distribution of COPD patients according to GOLD categories. Category B had the largest proportion of patients (41.3%), while category C had the smallest (7.5%). Table 2 (see PDF) presents the prevalence of anxiety across GOLD categories using GAD-7. The highest prevalence of anxiety was observed in GOLD category D (57.78%). Table 3 (see PDF) illustrates the prevalence of depression across GOLD categories using PHQ-9. GOLD category D had the highest prevalence of depression (68.89%). Table 4 (see PDF) shows the correlation of GOLD categories with anxiety and depression scores. A statistically significant weak positive correlation was observed between GOLD category and anxiety scores (ρ = 0.351, P < 0.01) and a moderate positive correlation with depression scores (ρ = 0.415, P < 0.01).

## Discussion:

This study demonstrates a high prevalence of anxiety (36.25%) and depression (44.38%) among COPD patients, with psychological morbidity increasing with disease severity. These findings align with previous studies showing frequent psychological distress in COPD patients. Anxiety and depression were more prevalent in GOLD category D patients, as noted by Qiu *et al.* who reported increased depressive symptoms with severe COPD due to persistent dyspnea and reduced functional capacity [8]. A significant positive correlation was found between GOLD category and anxiety scores (ρ = 0.351, P < 0.01), suggesting worsening disease severity heightens anxiety. Funk *et al.* also found a high burden of anxiety in advanced COPD, linked to fear of breathlessness and exacerbations [9]. Similarly, a moderate positive correlation was observed between GOLD category and depression scores (ρ = 0.415, P < 0.01), indicating a strong association between disease severity and depression. These findings align with Deliu *et al.* who identified a bidirectional relationship between COPD and depression, with each condition worsening the other [10]. Anxiety and depression in COPD are associated with poor treatment adherence, increased healthcare utilization and reduced participation in pulmonary rehabilitation [11]. Early identification and management of these comorbidities can improve treatment compliance and quality of life. Routine screening with tools like PHQ-9 and GAD-7, as used in this study, is recommended for early detection. Integrated care approaches that combine pulmonary and psychological management lead to better outcomes [12]. Untreated psychological distress increases the risk of COPD exacerbations and hospitalizations. Laurin *et al.* showed that psychological distress predicts exacerbation risk, emphasizing the need for comprehensive management [13].

## Conclusion:

Anxiety and depression are prevalent in COPD patients and increase with disease severity. A significant positive correlation between GOLD categories and psychological distress underscores the interplay between physical and mental health. Routine screening with tools like PHQ-9 and GAD-7, combined with integrated care, is crucial for improving treatment adherence and quality of life.

## References

[1] Rahi MS (2023). Advances in respiratory medicine..

[2] Rahi MS (2023). Adv Respir Med..

[3] Wang J (2021). Care Respir. Med..

[4] Kamariotou E (2025). Diseases..

[5] Siraj RA (2025). Medicina (Kaunas)..

[6] Agustí­ A (2023). Am J Respir Crit Care Med..

[7] Jelic I (2023). European Journal of Investigation in Health, Psychology and Education..

[8] Qiu CJ, Wu S (2024). World J Psychiatry..

[9] Funk GC (2009). Respir Res..

[10] Deliu A (2026). Medical Sciences..

[11] Volpato E (2021). Int J Chron Obstruct Pulmon Dis..

[12] Zhao J (2024). Neuropsychiatr Dis Treat..

[13] Laurin C (2012). Am J Respir Crit Care Med..

